# Achieving Efficient p‐Type Organic Thermoelectrics by Modulation of Acceptor Unit in Photovoltaic *π*‐Conjugated Copolymers

**DOI:** 10.1002/advs.202103646

**Published:** 2021-12-02

**Authors:** Junhui Tang, Jingjing Ji, Ruisi Chen, Yongkun Yan, Yan Zhao, Ziqi Liang

**Affiliations:** ^1^ Department of Materials Science Fudan University Shanghai 200433 China

**Keywords:** D−A copolymers, doping, molecular packing, p‐type thermoelectrics, semiconducting polymers

## Abstract

*π*‐Conjugated donor (D)−acceptor (A) copolymers have been extensively studied as organic photovoltaic (OPV) donors yet remain largely unexplored in organic thermoelectrics (OTEs) despite their outstanding mechanical bendability, solution processability and flexible molecular design. Importantly, they feature high Seebeck coefficient (*S*) that are desirable in room‐temperature wearable application scenarios under small temperature gradients. In this work, the authors have systematically investigated a series of D−A semiconducting copolymers possessing various electron‐deficient A‐units (e.g., BDD, TT, DPP) towards efficient OTEs. Upon p‐type ferric chloride (FeCl_3_) doping, the relationship between the thermoelectric characteristics and the electron‐withdrawing ability of A‐unit is largely elucidated. It is revealed that a strong D−A nature tends to induce an energetic disorder along the *π*‐backbone, leading to an enlarged separation of the transport and Fermi levels, and consequently an increase of *S*. Meanwhile, the highly electron‐deficient A‐unit would impair electron transfer from D‐unit to p‐type dopants, thus decreasing the doping efficiency and electrical conductivity (σ). Ultimately, the peak power factor (PF) at room‐temperature is obtained as high as 105.5 µW m^−1^ K^−2^ with an outstanding *S* of 247 µV K^−1^ in a paradigm OPV donor PBDB‐T, which holds great potential in wearable electronics driven by a small temperature gradient.

## Graphical abstract

Three *π*‐conjugated D−A copolymers with various acceptor (A) units and the same donor (D) unit are investigated to reveal the relationship between the thermoelectric characteristics and electron‐withdrawing ability of A‐unit. As a result, a peak power factor (PF) as high as 105.5 µW m^−1^ K^−2^ coupled with an outstanding Seebeck coefficient (*S*) of 247 µV K^−1^ is obtained in the paradigm PBDB‐T.

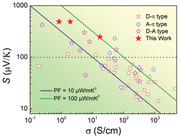

## 1 Introduction

Thermoelectric (TE) materials are promising green technology that can directly transform heat into electricity according to the Seebeck effect without any moving parts or noise.^[^ ^1^, ^2^, ^3^ ^]^ The dimensionless figure of merit (*zT*) is generally used to characterize the TE performance by an equation of *zT* = *S* ^2^ *σ*T/*κ* where *S*, *σ*, *κ*, and T represent the Seebeck coefficient (V K^−1^), electrical conductivity (S m^−1^), thermal conductivity (W m^−1^ K^−1^), and absolute temperature (K), respectively.^[^ ^4^, ^5^ ^]^ It is worth noting that *S* is proportional to the difference between transport energy level (*E*_T_) and Fermi level (*E*_F_) at a specific temperature. Besides, *σ* equals to the product of charge carrier mobility (*μ*), carrier density (*n*), and elementary charge (e), while *S* is adversely correlated to *n*.

The last decade has witnessed an extensive investigation of organic semiconductors (OSCs) in the field of thermoelectrics thanks to their facile solution‐processability at room temperature, flexible molecular design and in particular superior mechanical bendability compared to their inorganic counterparts.^[^ ^6^, ^7^, ^8^ ^]^ However, the comparatively low TE performance of OSCs largely retards their practical applications. Thus, it is of essential importance to improve the numerator of *zT* value, defined as power factor (PF = *S* ^2^ *σ*), in OSCs, since they typically show negligible total *κ* (<0.5 W m^−1^ K^−1^), including collective contributions from electrons (*κ*_e_) and lattice (*κ*_L_).^[^ ^9^ ^]^ Given the superior mechanical flexibility to their small‐molecule analogues, organic semiconducting polymers are endowed with the readily realized large‐area roll‐to‐roll printing techniques towards the applications in wearable electronics, such as miniature sensors, flexible thermoelectric generators, and so on.^[^ ^10^, ^11^, ^12^, ^13^ ^]^ The wearable application scenario usually features a small temperature gradient (e.g., <5 K) between hot and cold ends, which demands a high *S* to realize the remarkable voltage output.

p‐Type semiconducting polymers can be categorized into three main types according to their repeating units along the main chain. The first is so‐called D−*π* type that contains alternating donor (D) unit and *π*‐conjugated bridge, representative of homopolymer poly(3‐hexylthiophene) (P3HT) and poly(2,5‐bis(3‐tetradecylthiophen‐2‐yl)thieno[3,2‐b]thiophene) (PBTTT‐C_14_). Most attempts were focused on the *σ* enhancement by increasing carrier mobility through various film fabricating methods^[^ ^14^, ^15^ ^]^ or improving doping efficiency via exploiting novel doping methods while unveiling the underlying mechanisms.^[^ ^16^, ^17^, ^18^ ^]^ For instance, a vapor‐doping method was employed by Chabinyc and coworkers instead of the solution‐mixing method to increase the electrical properties of PBTTT‐C_14_ films thanks to the alignment of ordered domains in vapor‐doped thin films for efficient charge transport. The *S* was measured to be 42 µV K^−1^ with *σ* as high as 670 S cm^−1^.^[^ ^19^ ^]^ By conducting a thorough survey of the literature, it is found that most of well‐performing p‐type semiconducting polymers belong to D−*π* type. However, they generally display an outstanding *σ* yet an inferior *S* lower than 50 µV K^−1^ at a high carrier concentration. Considering that high *S* is required for the applications of polymeric TE materials in wearable devices, D−*π* copolymers seem difficult to satisfy this demand. The introduction of acceptor (A) moiety into the backbone yields the second A−*π* type copolymers when A units are paired with *π*‐bridges, which is best exemplified by poly(diketopyrrolopyrrole‐terthiophene) (PDPP‐3T).^[^ ^20^ ^]^ The third class of copolymers are comprised of alternate D and A units along the backbone, and such D−A type copolymers have been widely used as electron‐donor components in organic photovoltaics.^[^ ^21^ ^]^ Different from the D−*π* type copolymers, the intramolecular interactions between D and A units can effectively extend the *π*‐conjugation length and strengthen the hole‐transporting ability in the main chain and meanwhile enhance intermolecular *π*‐stacking.^[^ ^22^ ^]^ Many previous works were focused on enhancing the TE properties of D−A copolymers mainly by either side‐chain engineering or incorporation of halogen atom as side‐group in the backbone.^[^ ^23^, ^24^ ^]^ For example, Lee and coworkers reported that substituting normal alkyl side‐chains with sp^2^‐hybridized olefinic bis(alkylsulfanyl)methylene led to extended chain planarity and enhanced interchain packing. The modified copolymer PCPDTSBT showed one order of magnitude higher *σ* (2.13 S cm^−1^) coupled with *S* as high as 190.5 µV K^−1^ upon Lewis acid B(C_6_F_5_)_3_ doping.^[^ ^25^ ^]^ An alternate substitution by polar oligoethylene glycol as side‐group led to PCPDTSBT‐A, which presented significant self‐doping and greatly improved *σ* of 53.8 S cm^−1^ after sequential doping with 2,3,5,6‐tetrafluoro‐7,7,8,8‐tetracyanoquinodimethane (F_4_TCNQ).^[^ ^26^ ^]^ On the other side, Li and colleagues reported such a random copolymerization of D−D and D−A type moieties into the backbone, the former of which contributes to effective charge transfer while the latter ensures efficient carrier transport. The resulting optimal copolymer PDPP‐g_3_2T_0.3_ with finely tuned D/A unit ratios delivered an excellent PF as high as 110 µW m^−1^ K^−2^ with a moderate *S* of 56 µV K^−1^.^[^ ^27^ ^]^ In general, owing to the sufficient energetic disorder in their molecular structures, D−A copolymers exhibit a relatively higher *S* than D−*π* analogues.^[^ ^28^ ^]^ Nonetheless, a multitude of studies have been devoted to increasing the doping efficiency and hence *σ*, yet the methods of obtaining high *S* are rarely explored. Thus, we are dedicated in this work to enhancing *S* by cautiously selecting appropriate A units in the polymeric backbone while unveiling the influencing factors.

Herein, we used the same donor unit, benzodithiophene (BDT), to copolymerize with three different acceptor units and studied the relationship between the TE properties of the obtained D−A copolymers and the electron‐withdrawing ability of A‐units. Ferric chloride (FeCl_3_) was employed as the p‐type dopant and ultraviolet–visible–near infrared absorption spectroscopy was measured to confirm that all copolymers were successfully doped. The molecular packing motifs were characterized by grazing‐incidence wide‐angle X‐ray scattering (GIWAXS) profiles, in which PBDB‐T presented bimodal orientation and most compact packing behaviors. Thus, PBDB‐T exhibited the highest in‐plane charge transport, which was proved by the carrier mobility as obtained from the organic field‐effect transistor (OFET) devices. Furthermore, it was found that the stronger the electron‐withdrawing ability of A‐unit in the copolymers, the higher Seebeck coefficient yet lower electrical conductivity were achieved. Ultimately, the room‐temperature peak power factor as high as 105.5 µW m^−1^ K^−2^ was obtained in PBDB‐T at an optimal dopant concentration of 10 mmol L^−1^.

## 2 Results and Discussion

**Figure 1:**
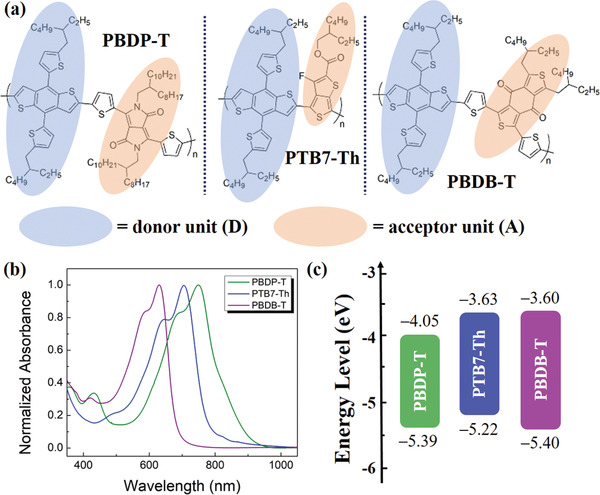
p‐Type D−A copolymers of PBDP‐T, PTB7‐Th, and PBDB‐T: a) molecular structures, b) ultraviolet–visible absorption spectra, and c) energy level diagrams. Note D = donor unit: BDT; A = acceptor unit: DPP > TT > BDD in electron‐withdrawing capability.

The semiconducting donor−acceptor (D−A) copolymers employed in this work are all based on the same donor moiety BDT and copolymerized with a series of acceptor units possessing various electron‐withdrawing abilities. As shown in **Figure** **1**a, the A unit of PBDP‐T, that is, poly{2,6′‐4,8‐di(5‐ethylhexylthienyl)benzo[1,2‐b;3,4‐b]dithiophene‐alt‐5,5″‐dioctyldodecyl‐3,6‐bis(5‐thiophen‐2‐yl)pyrrolo[3,4‐c]pyrrole‐1,4‐dione} is diketopyrrolopyrrole (DPP), which is more electron‐deficient than thieno[3,4‐b]thiophene (TT), the A unit of PTB7‐Th, that is, poly[4,8‐bis(5‐(2‐ethylhexyl)thiophen‐2‐yl)benzo[1,2‐b;4,5‐b″]dithiophene‐2,6‐diyl‐alt‐(4‐(2‐ethylhexyl)‐3‐fluorothieno[3,4‐b]thiophene)‐2‐carboxylate‐2‐6‐diyl)]. The frontier molecular orbitals of D−A copolymers are determined together by D and A units, in which the highest occupied molecular orbital (HOMO) and lowest unoccupied molecular orbital (LUMO) energy levels depend mainly on the D and A moiety, respectively. Thus, the semiconducting polymers with strong D−A characteristics generally display low‐lying LUMO and high‐lying HOMO levels, leading to relatively narrow optical bandgaps (*E*_g_s). Since the benzodithiophene‐dione (BDD) unit in PBDB‐T, that is, poly[(2,6‐(4,8‐bis(5‐(2‐ethylhexyl)thiophen‐2‐yl)‐benzo[1,2‐b:4,5‐b’]dithiophene))‐alt‐(5,5‐(1’,3’‐di‐2‐thienyl‐5’,7’‐bis(2‐ethylhexyl)benzo[1’,2’‐c:4’,5’‐c’]dithiophene‐4,8‐dione)] retains the least electron‐withdrawing capability, given that the D unit is the same in three polymers, PBDB‐T evidently displays the shallowest LUMO level (−3.60 eV) and the largest *E*_g_ (1.80 eV) as shown in Figure 1c. It is noteworthy that the HOMO energy levels of copolymers were obtained by cyclic voltammetry (CV) measurements through the equation *E*_HOMO_ = −(4.35 + *E*_ox_^onset^) eV and all the reduction waves are undetectable due to the electron‐rich characteristics of the copolymers. The LUMO energy levels were achieved by adding up *E*_HOMO_ and the optical bandgaps extracted from the onset wavelengths of absorption spectra as shown in Figure 1b. The LUMO and HOMO energy levels of PBDP‐T and PTB7‐Th are estimated to be −4.05, −5.39, −3.63, and −5.22 eV, respectively, as shown in Figure 1c and Table S1, Supporting Information. The relatively shallower LUMO energy level of PTB7‐Th than PBDP‐T and PBDB‐T may be due to the absence of *π*‐unit thiophenes in the backbone, which would strengthen the average electron‐donating capability of D‐units to some extent because thiophenes can also serve as donors yet possessing inferior electron‐donating ability. As a result, the *E*_g_ of PBDP‐T with the strongest electron‐withdrawing unit DPP is the smallest (1.34 eV) among the three D−A copolymers.

**Figure 2:**
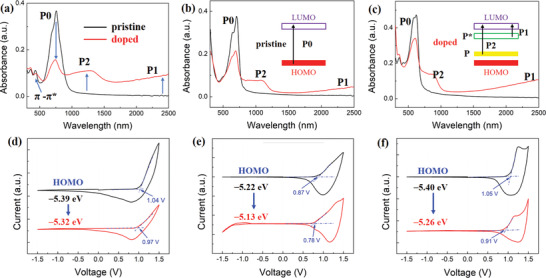
UV–vis–NIR absorption spectra of pristine and FeCl_3_ (10 mM) doped a) PBDP‐T, b) PTB7‐Th, and c) PBDB‐T thin films. The insets are the energy bands and corresponding absorption peaks at pristine and doped state, respectively. Cyclic voltammograms of pristine and FeCl_3_ (10 mM) doped d) PBDP‐T, e) PTB7‐Th, and f) PBDB‐T thin films deposited on the working electrode immersed in 0.1 M n‐Bu_4_PF_6_ acetonitrile solution scanned at 50 mV s^−1^.

In order to investigate the p‐doping behavior of all copolymers, ultraviolet–visible–near infrared (UV–vis–NIR) absorption spectroscopy was employed. For pristine films, the maximum absorption peak of PBDP‐T is explicitly red‐shifted relative to that of PBDB‐T and PTB7‐Th, due to the significantly reduced *E*_g_, as shown in **Figure** **2**a–c. The shoulder peak of PBDB‐T ≈600 nm is the most noticeable among the three copolymers, indicative of better molecular packing motifs, while there is no obvious optical absorption above 1000 nm in the pristine films. All doped films were immersed in 10 mm FeCl_3_/acetonitrile solution for 1 min with stirring to trigger the doping process. The absorption of the pristine polymers bleaches in the visible range and new bands appear in the NIR region, which can be ascribed to the formation of (bi)polaronic species as shown in Figure 2a–c and Figure S2, Supporting Information, suggesting an occurrence of charge transfer between p‐dopants and polymer *π*‐backbones. In details, P0 refers to the broad interband transition of neutral polymers and the corresponding absorption bleaching is owing to the formation of free radical cations or cations generated by the reactions that FeCl_3_ dopants provide holes to the copolymers. The new absorption bands of P2 and P1 upon doping are assigned to polaron transitions and polaron absorption peaks, respectively. Meanwhile, although the ratio of polaron to the neutral absorption peak area of PBDP‐T is evidently larger than that of PTB7‐Th and PBDB‐T, this value is unable to evaluate the doping levels among different copolymers given that its extensive impacting factors including molecular packing, the effective *π*‐conjugation length, and so on.^[^ ^29^ ^]^

The aromatic structures of the three copolymers are reorganized into quinoid structures, which are the features of the (bi)polaronic species, upon doping. Thus, the polaron bands are prone to show an energy level higher than the HOMO energy level of the pristine copolymers.^[^ ^29^ ^]^ In order to gain further insights into the variation of energy levels upon p‐doping, CV measurements were performed and the profiles are shown in Figure 2d–f. In the pristine state, the oxidation waves of PBDP‐T, PTB7‐Th, and PBDB‐T are 1.04, 0.87, and 1.05 V, respectively. After p‐doping, the onsets of oxidation potentials tend to decrease in all these copolymers, indicative of an upshift of HOMO energy levels. Among them, PBDB‐T exhibits the largest shift (0.14 eV) of HOMO energy levels from −5.40 to −5.26 eV, acting as an evidence of the highest doping efficiency achieved in PBDB‐T.

Besides, X‐ray photoelectron spectroscopy (XPS) measurements were conducted to acquire the doping level and dopant content in the 10 mM FeCl_3_ doped polymer films. As shown in Figure S3 and Table S2, Supporting Information, the broad Cl 2p peak can be split into two narrower peaks ≈200 and ≈202 eV, respectively, in which the former corresponds to Cl—Fe bonding in ${\mathrm{FeCl}}_{4}^{-}$ anion and FeCl_2_ and the latter is assigned to Cl—C bonding. The Cl—C bonding is formed during the doping process of $2\mathrm{FeC}l_{3}+\;e^{-}\;\to {\mathrm{FeCl}}_{4}^{-}+\mathrm{FeC}l_{2}$, in which ${\mathrm{FeCl}}_{4}^{-}$ act as counterions of polymer radical cations. Thus, the up‐field shift of the atomic binding energy indicates the higher Cl—C bonding proportion, which can be translated into higher doping level in PBDB‐T than PTB7‐Th and PBDP‐T.

**Figure 3:**
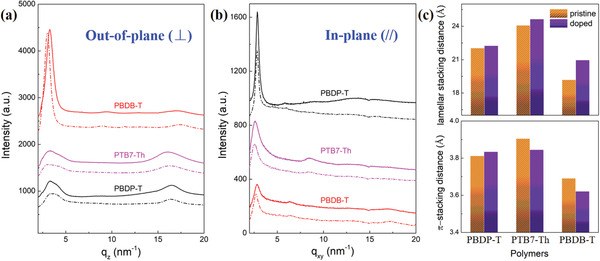
GIWAXS profiles of pristine (solid line) and doped (dash‐dot line) polymers. a) Out‐of‐plane (⊥) and b) in‐plane (//) patterns. c) Lamellar stacking and *π*–*π* stacking distances as extracted from both (a) and (b).

Next, GIWAXS characterization was carried out to unravel the molecular packing motifs of polymers and accordingly comprehend how the doping process influences the charge transport behaviors. The out‐of‐plane (⊥) and in‐plane (//) scattering profiles and the corresponding two‐dimensional (2D)‐GIWAXS images are shown in **Figure** **3**a,b and Figures S4−S6, Table S3, Supporting Information, respectively. It is unveiled that pristine PBDP‐T exhibits a high degree of ordering along the *q*_xy_ direction with face‐on orientations, which may be due to the ultrahigh molecular weight as shown in Figure S1 and Table S1, Supporting Information. The lamellar packing (100) distance is calculated from the peak to be 22.04 Å, corresponding to *q*_xy_ = 0.29 Å^−1^, along with a *π*‐stacking (010) distance of 3.81 Å (*q*_z_ = 1.65 Å^−1^). In contrast, PTB7‐Th film also shows face‐on orientations but has a larger lamellar distance of 24.08 Å (*q*_xy_ = 0.26 Å^−1^) and a longer *π*–*π* stacking distance of 3.91 Å (*q*_z_ = 1.61 Å^−1^). Distinctly, PBDB‐T exhibits totally different molecular packing behaviors, which is called bimodal orientation.^[^ ^30^ ^]^ In other words, PBDB‐T shows both face‐on and edge‐on orientations, although with the former dominant, endowing it with best in‐plane charge transport among the three copolymers. The calculated lamellar packing and *π*–*π* stacking distances along the out‐of‐plane direction are as small as 19.19 (*q*_z_ = 0.33 Å^−1^) and 3.69 Å (*q*_z_ = 1.70 Å^−1^), respectively, suggesting that PBDB‐T has remarkably compact molecular packing compared to both PBDP‐T and PTB7‐Th. Interestingly, the absence of *π*‐bridges (thiophenes) in PTB7‐Th between the alternating rigid D and A units may hinder the side‐chains from packing densely to the *π*‐backbone and hence the largest lamellar and *π*‐stacking distances are obtained.

After doping by FeCl_3_, the lamellar stacking distance of PBDP‐T is slightly increased to 22.25 Å and the difference before/after doping (namely, Δ*d*_100_) is determined to be 0.21 Å, while the *π*‐stacking arises marginally to 3.83 Å with Δ*d*_010_ = 0.02 Å. However, the *π*‐stacking distances of doped PTB7‐Th and PBDB‐T are both reduced down to be 3.85 (Δ*d*_010_ = −0.06 Å) and 3.62 Å (Δ*d*_010_ = −0.07 Å), respectively, which can be attributed to the enhanced interactions between adjacent polymer chains upon doping. Meanwhile, the lamellar packing distances of doped PTB7‐Th and PBDB‐T both significantly are increased up to 24.64 (Δ*d*_100_ = 0.56 Å) and 20.95 Å (Δ*d*_100_ = 1.76 Å), respectively. This indicates that the dopants mainly reside in the side‐chain regions and have little negative impact on *π*–*π* transport channels. It is worth noting that the significantly larger increase in lamellar packing distance of PBDB‐T than that of PBDP‐T may imply a stronger interaction between p‐dopant and side chains of PBDB‐T versus PBDP‐T, suggestive of a higher doping level in PBDB‐T. Crystal coherence length (CCL) and para‐crystalline disorder (*g*) of (100) and (010) diffraction peaks were calculated to compare the crystallinities of intrinsic and p‐doped D−A copolymers as shown in Tables S4 and S5, Supporting Information. For each copolymer, the CCL of both (100) and (010) diffraction peaks increase and the corresponding *g* decreases with the introduction of p‐dopants, indicating their lamellar and *π*–*π* packing crystallinities both increase. This phenomenon is favorable for the charge transport of copolymers after doping and may boost the carrier mobility.

**Figure 4:**
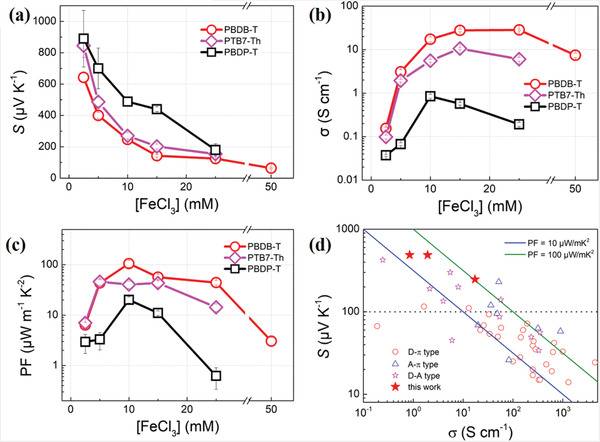
Thermoelectric characteristics of doped polymer thin films as a function of dopant concentration. a) Seebeck coefficient (*S*), b) electrical conductivity (*σ*), c) power factor (PF), and d) their comparison with literature results among different types of p‐type semiconducting polymers.^[^ ^14^, ^15^, ^16^, ^17^, ^18^, ^19^, ^20^, ^23^, ^24^, ^25^, ^26^, ^27^, ^32^, ^33^, ^34^, ^35^, ^36^, ^37^, ^38^, ^39^, ^40^, ^41^, ^42^, ^43^, ^44^, ^45^, ^46^, ^47^, ^48^, ^49^, ^50^, ^51^, ^52^, ^53^, ^54^ ^]^

To elucidate how the choice of A‐moiety correlates with the electrical properties, we doped all polymers by using FeCl_3_ under various concentrations in solution. All thin films were immersed in FeCl_3_/CH_3_CN solution with concentrations ranging from 2.5 to 50 mM for 1 min (see details in the Experimental Section). It is found that the TE parameters could be effectively varied by modulation of A units. As shown in **Figure** **4**a, all copolymers show positive *S*, indicating that holes are the majority carriers. The *S* of PBDP‐T continuously decreases from 890 to 180 µV K^−1^ with increasing FeCl_3_ concentration ranging from 2.5 to 25.0 mm. The same decreasing trend is also found in the other two copolymers, owing to the elevation of doping level and carrier concentration with increasing [FeCl_3_]. It is noteworthy that at each dopant concentration, *S* monotonically increases since the electron‐withdrawing ability of A unit is enhanced by switching from BDD, TT to DPP unit. As shown in Figure 4b, in contrast, *σ* first rises and then drops with the increase of dopant concentration. The former is caused by the dominance of the uplift of carrier concentration while the latter is ascribable to the pronounced decrease of carrier mobility owing to defects induced by excessive dopants. Ultimately, the maximum *σ* values of PBDP‐T, PTB7‐Th, and PBDB‐T are determined to be 0.85, 10.6, and 28.4 S cm^−1^ at various dopant concentrations of 10, 15, and 25 mm, respectively. The highest *σ* of PBDB‐T results from not only the comparatively high p‐doping efficiency as evidenced by CV and XPS measurements but also the compact stacking of polymer molecules as indicated in Figure 3. Interestingly, at each dopant concentration, *σ* monotonically decreases as the electron‐withdrawing ability of acceptor moiety enhances, which is diametrically opposite to the trend in *S*. Owing to the inactivity of A units upon p‐doping, the positive charged polarons of the D–A copolymers are predominantly localized on the D units.^[^ ^28^, ^31^ ^]^ As a result, with the increase of the electron deficient property of A units, the radical cations are less delocalized along the polymer backbone, which is unfavorable for charge transport and the *σ*. Of course, the D–A characteristics is not the only impacting factor of the delocalization length, the coplanarity of polymers is also closely related to it and the compact molecular packing in PBDB‐T may further facilitate delocalization. On the other side, the strong electron‐withdrawing ability of the A unit would compete with p‐dopants to attract electrons from the D unit, thus impairing the hole transfer process from p‐dopants to D units, resulting in a low p‐doping efficiency and hence the *σ*. Meanwhile, the strong electron‐withdrawing capability of A moiety could strengthen the energetic disorder along the polymer chains and tuning the shape of density‐of‐states (DOS), thus enlarging the value of *E*_F_ − *E*_T_, which is proportional to *S*.^[^ ^27^ ^]^ Accordingly, the peak room‐temperature PFs of PBDP‐T, PTB7‐Th, and PBDB‐T are acquired to be as high as 20.1, 46.0, and 105.5 µW m^−1^ K^−2^ at [FeCl_3_] = 10, 5, and 10 mm, respectively, as shown in Figure 4c. When comparing with the literature results of D–*π*,^[^ ^14^, ^15^, ^16^, ^17^, ^18^, ^19^, ^32^, ^33^, ^34^, ^35^, ^36^, ^37^, ^38^, ^39^, ^40^, ^41^, ^42^, ^43^, ^44^, ^45^, ^46^, ^47^, ^48^ ^]^ A–*π*,^[^ ^20^, ^49^, ^50^, ^51^ ^]^ and D–A^[^ ^23^, ^24^, ^25^, ^26^, ^27^, ^52^, ^53^, ^54^ ^]^ type copolymers as summarized in Figure 4d, it has been rarely reported with *S* values over 100 µV K^−1^, most of which were obtained by those D–A copolymers. This work achieves an impressively high PF over 100 µW m^−1^ K^−2^ while preserving a superior *S* beyond 200 µV K^−1^ as shown in Table S6, Supporting Information, holding great promise in wearable skin electronics, taken as one example.

To gain an insight into the charge transport behavior among these D−A copolymers, the temperature‐dependent TE characterstics were investigated. As shown in Figure S7, Supporting Information, three copolymers present similar trends in *S*, *σ*, and PF upon elevated temperature. The *S* shows a monotonous increase when the temperature is raised from 303−333 K. On the other hand, the *σ* first rises and then decreases with elevated temperature in all three copolymers. The increase of *σ* within a lower temperature range is attributed to the thermally activated electrical conductivity while the subsequent decline is due to de‐doping of heavily doped polymers at higher temperature. The noticeable larger slope of *S*/T at higher temperature confirms an occurrence of de‐doping to some degree. Ultimately, the highest PFs of PBDP‐T, PTB7‐Th, and PBDB‐T are obtained as 25.3, 44.6, and 113.6 µW m^−1^ K^−2^ at 318.0, 327.3, and 322.5 K, respectively.

**Figure 5:**
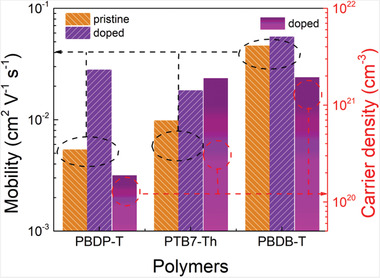
Charge carrier mobilities of pristine and FeCl_3_ (10 mm) doped D–A copolymers extracted from the saturated region of the transfer curves of OFET devices. The charge carrier density of FeCl_3_ (10 mm) doped D–A copolymers is calculated by an equation of *n* = *σ*/e*µ*.

Finally, we attempted to better understand the factors influencing the TE properties of D–A copolymers bearing various A units and the underlying mechanisms that led to higher PFs in PBDB‐T than those in PBDP‐T and PTB7‐Th. Thus, we turned to construct bottom gate–bottom contact OFET devices to acquire hole mobility (*μ*_h_) values. As shown in Figures S8–S10, Supporting Information, PBDP‐T displays the lowest off‐state current (9.0 × 10^−10^ A) among PTB7‐Th (4.7 × 10^−9^ A) and PBDB‐T (1.8 × 10^−8^ A). As shown in **Figure** **5**, the *μ*_h_ values were extracted from the saturation region of the transfer curves, among which PBDB‐T exhibits the highest 0.046 cm^2^ V^−1^ s^−1^, in consistence with the GIWAXS results. By measuring the *μ*_h_ values of these copolymers upon doping with FeCl_3_ at 10 mm, it is discovered that the immersion doping method we employed in this work would not pronouncedly disturb the molecular packing of polymers as evidenced by the variation of CCL and *g* factor in Tables S4 and S5, Supporting Information. Similar phenomenon has been reported that the reduction of energy disorder, caused by the decrease of energy barriers between Coulomb traps, led to the increase of carrier mobility upon doping.^[^ ^55^ ^]^ The *σ* values of D–A copolymers doped at 10 mm FeCl_3_ in Figure 4 were used to estimate the charge carrier density. It is noteworthy that the *σ* consisted of contributions from both holes and electrons, thus the *n* value calculated by *σ* and *μ*_h_ is over‐estimated to some extent. As a result, PBDP‐T shows the lowest *n* value of 1.88 × 10^20^ cm^−3^ whereas PBDB‐T displays the highest 1.94 × 10^21^ cm^−3^. Thus, the D–A copolymer with the least strong electron‐withdrawing A unit exhibits the best doping efficiency and *σ*, which is consistent with the above analyses. Moreover, as shown in Table S2, Supporting Information, the content of Fe is much lower in 10 mm FeCl_3_ doped PBDB‐T than that of PTB7‐Th and PBDP‐T. By combining with the results of highest *σ* and *n* as obtained in 10 mm FeCl_3_ doped PBDB‐T, the doping efficiency is significantly higher in PBDB‐T than the other two copolymers. Consequently, the acceptor strength itself largely determines the available doping level and charge density. In addition, *S*, which is strongly dependent on the doping level, is influenced to a great extent by the electron‐withdrawing ability of A unit. Therefore, both *S* and *σ* are closely correlated to the electron‐negativity of A moiety or namely, the D−A characteristics. It is noted that the *n* of PTB7‐Th and PBDB‐T is very close to each other (see Table S6, Supporting Information), which is exactly consistent with the similar *S* of these two copolymers at an FeCl_3_ concentration of 10 mm. Despite PBDB‐T displays the highest carrier mobility among the three copolymers, the *μ*_h_ still has sufficient room to be improved by further modification to increase the coplanarity, for instance, which would greatly facilitate charge transport and hence the *σ*.

## 3 Conclusions

In sum, we have employed the donor unit BDT to copolymerize with various acceptor units with enhanced electron‐withdrawing ability from BDD, TT to DPP. Among these three copolymers, PBDB‐T presents the most compact molecular packing and hence the highest carrier mobility is acquired as 0.046 cm^2^ V^−1^ s^−1^. Upon p‐doping, *σ* is improved with a decrease of electron‐withdrawing ability of acceptor units due to significantly enhanced charge transfer and increased carrier density. The *S* shows the opposite trend of *σ* owing to the enlarged energetic disorder in copolymers with strong D–A characteristics. Ultimately, the copolymer PBDB‐T displays a room‐temperature peak PF as high as 105.5 µW m^−1^ K^−2^ along with *S* of 247 µV K^−1^ at an optimal FeCl_3_ concentration of 10 mm. Of critical importance is the D–A copolymer with concurrent superior PF and ultrahigh *S*, which can be achieved via a judicious choice of D and A moieties, is an ideal candidate to fabricate wearable thermoelectric generators or sensors.

## 4 Experimental Section

### Chemicals and Materials

All monomers used in this work including DPP, BDD, and BDT and polymer PTB7‐Th were purchased from Derthon Optoelectronics Materials Science Technology Co., Ltd. Dopant anhydrous FeCl_3_ (≥97.0%) and solvent chlorobenzene (≥99.5%) were purchased from Sinopharm Chemical Reagent Co., Ltd. All chemicals and reagents were used as received and without any further purification.

### Synthesis of PBDB‐T

The monomer BDD (153.30 mg, 0.20 mmol), BDT (180.91 mg, 0.20 mmol), Pd_2_(dba)_3_ (3.66 mg, 0.004 mmol), and P(*o*‐tolyl)_3_ (4.87 mg, 0.016 mmol) were added into the tube, followed by adding 6 mL of anhydrous chlorobenzene into the mixture. The solution was stirred at 100 °C in an oil bath for 18 h under N_2_ atmosphere. The reaction was not terminated until the mixed solution was a gel. Then the mixture was cooled to room temperature and solvent methanol was added to filter. The precipitate was collected and the polymer was purified sequentially by Soxhlet extraction with methanol, petroleum ether, acetone, dichloromethane, and chloroform for 24 h, respectively. After drying, a dark solid polymer was obtained (190 mg, yield: 80%). GPC: *M*_n_ = 26.93 kDa, *M*_w_ = 40.75 kDa, PDI = 1.51; Anal. calcd for chemical formula: C_68_H_78_O_2_S_8_, C, 69.04; H, 6.60; S, 21.66. Found: C, 65.02; H, 6.90; S, 18.79.

### Synthesis of PBDP‐T

To obtain PBDP‐T, the synthesis and purification procedures were almost the same as those of PBDB‐T except for the amount of the monomers. The specific amount is as follows: BDT (180.91 mg, 0.20 mmol), DPP (203.84 mg, 0.20 mmol), Pd_2_(dba)_3_ (3.66 mg, 0.004 mmol), and P(*o*‐Tolyl)_3_ (4.87 mg, 0.016 mmol). A dark solid polymer was obtained (244 mg, yield: 85%). GPC results: *M*_n_ = 91.01 kDa, *M*_w_ = 262.00 kDa, PDI = 2.88; Anal. calcd for chemical formula: C_88_H_126_O_2_S_6_N_2_, C, 73.64; H, 8.79; S, 13.39; N, 1.95. Found: C, 72.01; H, 8.58; S, 12.93; N, 2.02.

### Fabrication of Copolymer Thin Films and TE measurements

The glass substrates were first cut into rectangle shape with a size of 15 mm × 12 mm. Then they were sonicated successively with deionized water, acetone, and iso‐propanol and dried with argon flow, followed by treating with oxygen plasma for 30 min. Then copolymer solutions of PBDP‐T, PTB7‐Th, and PBDB‐T in chlorobenzene (10 mg mL^−1^) were spin‐coated at a speed of 2000 rpm for 1 min and annealed at 100 °C for 10 min. For doped polymer thin films, the pre‐annealed film was immersed into FeCl_3_/acetonitrile solutions of various concentrations for 1 min and then annealed at 100 °C for 15 min to remove the residual solvent. The thicknesses of the copolymer thin films were measured to be 40–100 nm by profilometer. A four‐probe technique was used to measure the electrical conductivity on a multimeter (Keithley 2010) and a source meter (Keithley 2400). The Seebeck coefficient was measured by heating one resistor block while simultaneously measuring the generated temperature gradient (Δ*T*) and thermoelectric voltage (Δ*V*).

### Fabrication and Measurement of OFET Devices

Bottom gate–bottom contact OFETs were fabricated on highly doped Si wafers with a 300 nm SiO_2_ layer. Source and drain electrodes of 30 nm thick Au were patterned by photolithography onto the SiO_2_/Si wafers with channel length (*L*) of 20 µm and width (*W*) of 1400 µm. The substrates were first cleaned with piranha solution (volume mixing ratio: H_2_SO_4_:H_2_O_2_ = 2:1) for 20 min, then ultrasonically cleaned in deionized water and ethanol for 10 min each, and then baked in oven at 70 °C for 20 min. The substrates were placed in a petri dish with a small drop of octadecyltrichlorosilane (OTS) in the center and were treated in a vacuum oven at 120 °C for 3 h, followed by ultrasonically cleaned in hexane, ethanol, and chloroform for 5 min each. Then copolymer solutions of PBDP‐T, PTB7‐Th, and PBDB‐T in chlorobenzene (10 mg mL^−1^) were spin‐coated at a speed of 2000 rpm for 1 min on top of the OTS treated substrate and annealed at 100 °C for 10 min. For doped polymer‐based OFET devices, the pre‐annealed device was immersed into 10 mm FeCl_3_/acetonitrile solution for 1 min and then annealed at 100 °C for 15 min. All the measurements were performed immediately after the device fabrication was finished. The electrical characteristics were measured using a Keithley 4200A‐SCS Parameter analyzer. The saturation mobility was calculated using the transistor equation: *I*_DS_ = *W* / 2L*C_i_ *μ*(*V*_G_−*V*_T_)^2^, in which *C*_i_ (11.5 nF cm^−2^) is the unit area capacitance of the SiO_2_ dielectric; *I*_DS_ refers to the drain current; *V*_G_ and *V*_T_ represent the gate voltage and the threshold voltage, respectively.

### Characterization and Measurements

High‐temperature gel‐permeation chromatography (GPC) was conducted on a ShimadzuSIL‐20A liquid chromatography instrument using 1,2,4‐trichlorobenzene (TCB) as eluent at 150 °C with polystyrenes as standard. CV measurements were recorded with a three‐electrode cell under the ambient conditions in an anhydrous acetonitrile solution of tetran‐butylammonium hexafluorophosphate (0.1 m). A platinum disk electrode, platinum wire, and Ag/AgCl electrode were used as a working electrode, a counter electrode, and a reference electrode, respectively. The films of copolymers were drop‐casted on the surface of platinum disk electrode. The CV curves were scanned at 50 mV s^−1^ and calibrated using ferrocene/ferrocenium (Fc/Fc^+^) redox couple as an external standard, which were conducted at the same conditions as other samples. GIWAXS profiles were recorded at beamline BL14B1 of the Shanghai Synchrotron Radiation Facility (SSRF) at a wavelength of 1.2398 Å. UV–vis–NIR spectra were measured by HITACHI U‐4100 Spectrophotometer. X‐ray photoelectron spectroscopy (XPS) measurements were conducted with an X‐ray photoemission spectroscope (PHI5300), all the peaks were calibrated by C1s.

### Statistical Analysis

All experiments were performed at least twice with similar results. Data shown in Figure 4 are the average values ± standard deviations. Statistical tests were two‐sided if not mentioned otherwise. Statistical analysis was performed using a software of OriginPro, version 8.5.1.

## Conflict of Interest

The authors declare no conflict of interest.

## Supporting information

Supporting Information

## Data Availability

Research data are not shared.
