# Supplementary material for: Achieving Efficient p‐Type Organic Thermoelectrics by Modulation of Acceptor Unit in Photovoltaic π‐Conjugated Copolymers
Source: Adv Sci (Weinh). 2021 Dec 2;9(4):2103646. doi: 10.1002/advs.202103646 (PMC8811840; doi:10.1002/advs.202103646)
Supplement: Supplementary file 1 — Supporting Information [file ADVS-9-2103646-s001.pdf]

WILEY-VCH

ADVANCED SCIENCE
Open Access

Supporting Information

for *Adv. Sci.*, DOI: 10.1002/advs.202103646

Achieving Efficient p-Type Organic Thermoelectrics by Modulation of Acceptor Unit in Photovoltaic π-Conjugated Copolymers

*Junhui Tang, Jingjing Ji, Ruisi Chen, Yongkun Yan, Yan Zhao, and Ziqi Liang**

Supporting Information

Achieving Efficient p-Type Organic Thermoelectrics by Modulation of Acceptor Unit in Photovoltaic π-Conjugated Copolymers

*Junhui Tang, Jingjing Ji, Ruisi Chen, Yongkun Yan, Yan Zhao, and Ziqi Liang**

[*]Prof. Z. Liang, Dr. Y. Zhao, J. Tang, J. Ji, R. Chen, Y. Yan
Department of Materials Science, Fudan University
Shanghai 200433, China
Email: zqliang@fudan.edu.cn

Results

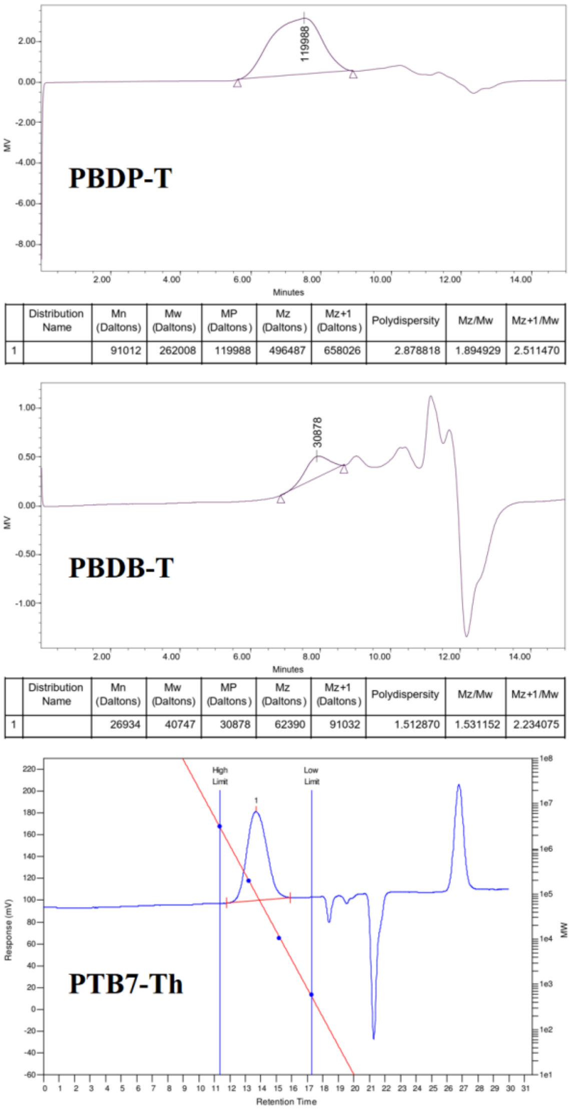

	Distribution Name	Mn (Daltons)	Mw (Daltons)	MP (Daltons)	Mz (Daltons)	Mz+1 (Daltons)	Polydispersity	Mz/Mw	Mz+1/Mw
1		91012	262008	119988	496487	658026	2.878818	1.894929	2.511470

	Distribution Name	Mn (Daltons)	Mw (Daltons)	MP (Daltons)	Mz (Daltons)	Mz+1 (Daltons)	Polydispersity	Mz/Mw	Mz+1/Mw
1		26934	40747	30878	62390	91032	1.512870	1.531152	2.234075

MW Averages

Peak No	Mp	Mn	Mw	Mz	Mz+1	Mv	PD
1	104446	60792	122308	216036	337696	111322	2.01191

Figure S1. GPC traces of PBDP-T, PBDB-T, and PTB7-Th. The molecular weight was evaluated with tricholorobenzene as eluent at 150 °C.

Table S1. Optical and electrochemical properties of three D−A copolymers

Copolymer	M_n (kDa)	PDI	λ_{max}^{film} (nm)	λ_{onset}^{film} (nm)	E_g^{opt} (eV)	LUMO (eV)	HOMO (eV)
PBDP-T	91.0	2.88	748	929	1.34	–4.05	–5.39
PTB7-Th	60.8	2.01	707	780	1.59	–3.63	–5.22
PBDB-T	26.9	1.51	628	689	1.80	–3.60	–5.40

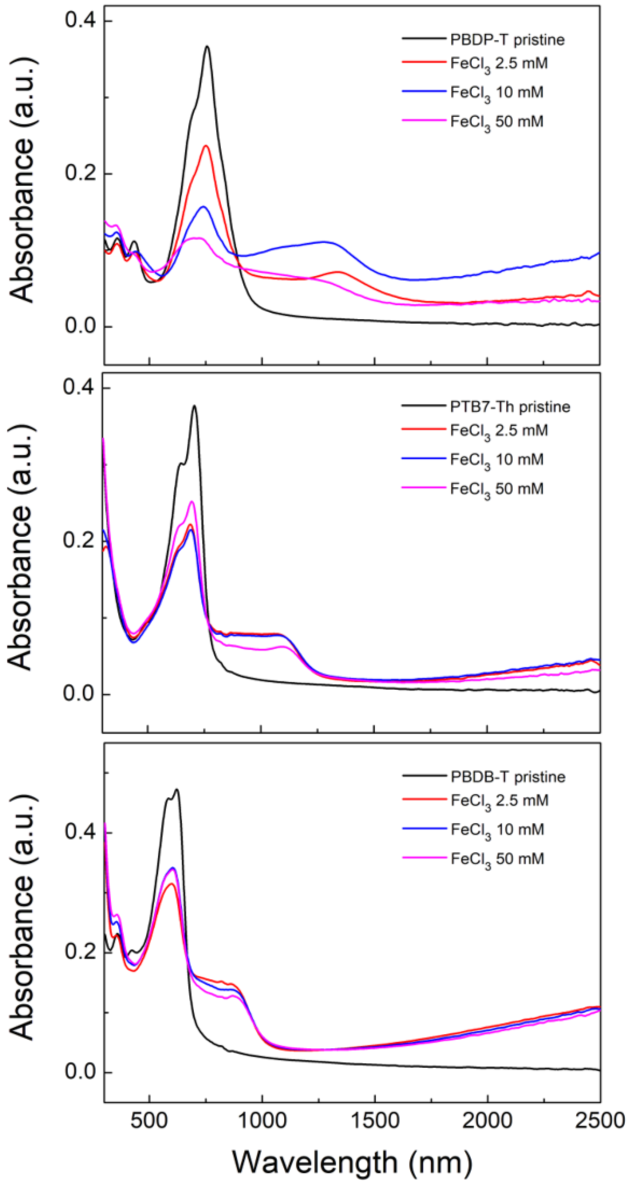

Figure S2. UV–vis–NIR spectra of pristine and doped copolymer thin films at different $FeCl_3$ concentrations.

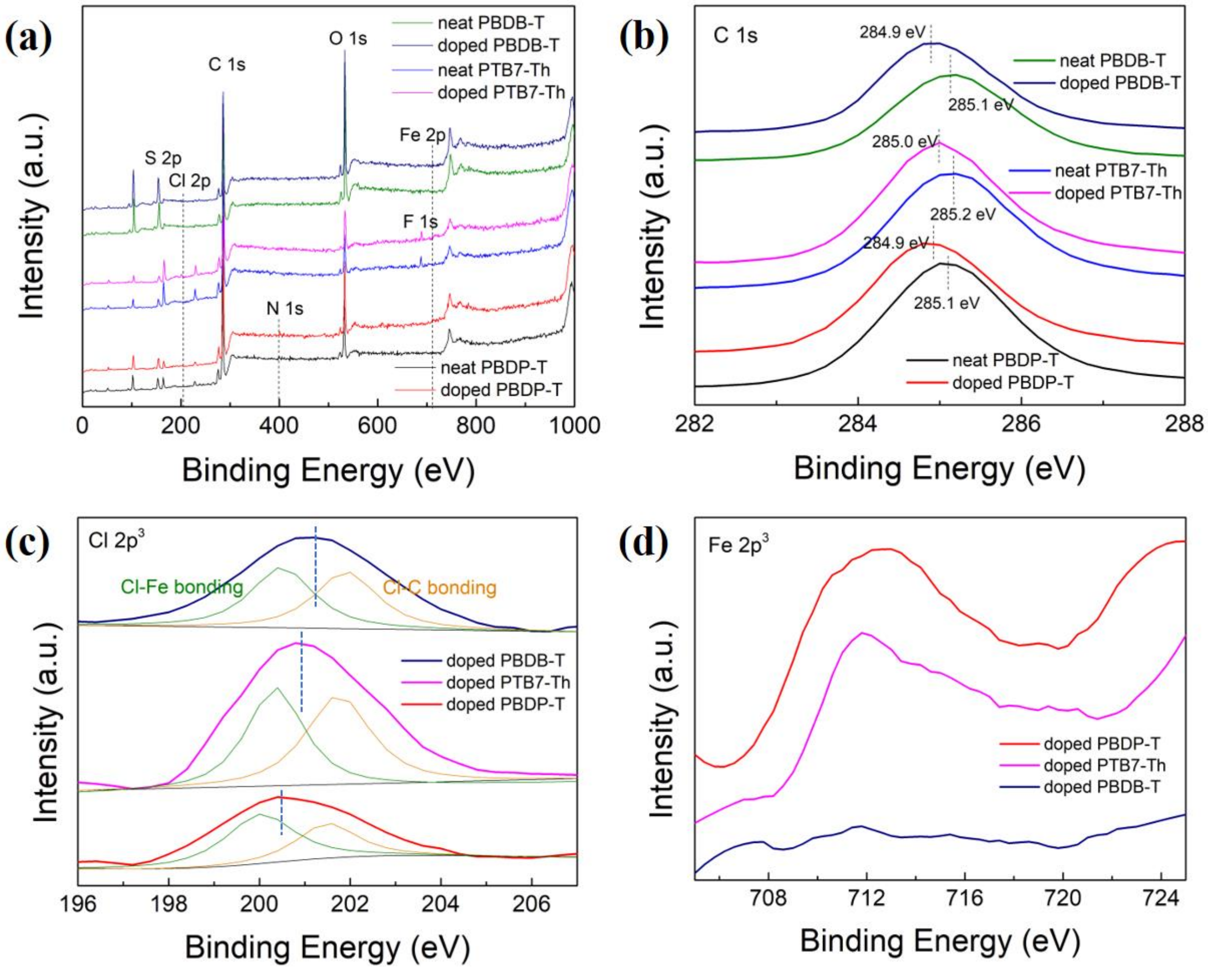

Figure S3. (a) XPS spectra of copolymers before and after $FeCl_3$ doping. (b) C 1s, (c) Cl 2p and (d) Fe 2p XPS spectra of PBDP-T, PTB7-Th and PBDB-T polymers.

Table S2. Molar ratio of elements in copolymers before and after doping (10 mM $FeCl_3$) retrieved by XPS

Sample	C (%)	N (%)	O (%)	F (%)	S (%)	Cl (%)	Fe (%)
neat PBDP-T	82.00	1.09	13.57	0.00	3.34	0.00	0.00
doped PBDP-T	82.22	0.97	13.85	0.00	2.07	0.37	0.52
neat PTB7-Th	82.71	0.23	9.41	1.16	6.49	0.00	0.00
doped PTB7-Th	81.95	0.61	9.25	1.11	5.92	0.74	0.42
neat PBDB-T	70.09	0.27	28.38	0.00	1.25	0.00	0.00
doped PBDB-T	69.37	0.49	27.86	0.00	1.48	0.65	0.16

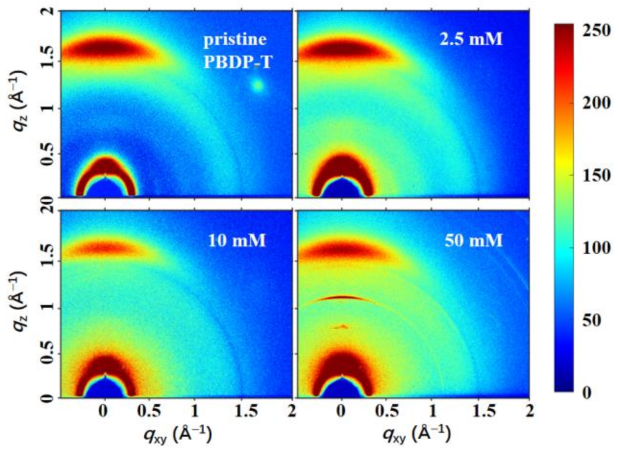

Figure S4. 2D GIWAXS images of pristine and doped PBDP-T thin films at various $FeCl_3$ concentrations.

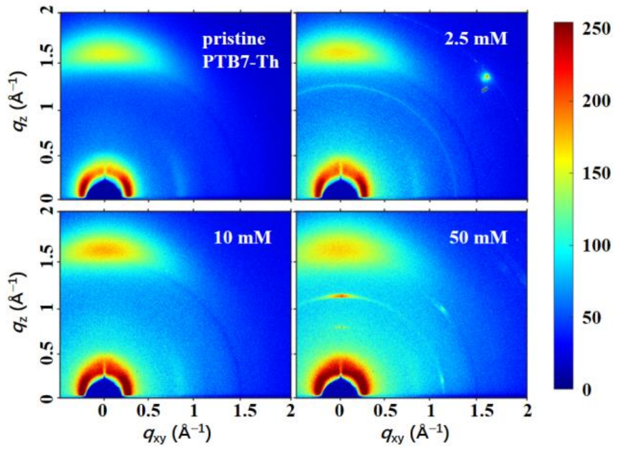

Figure S5. 2D GIWAXS images of pristine and doped PTB7-Th thin films at various $FeCl_3$ concentrations.

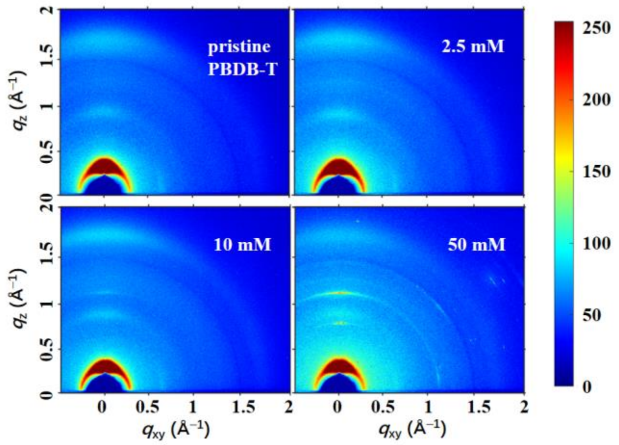

Figure S6. 2D GIWAXS images of pristine and doped PBDB-T thin films at various $FeCl_3$ concentrations.

Table S3. Lamellar and π–π stacking distances of pristine and doped copolymers

Polymer	$[FeCl_3]$ (mM)	q_{100} (nm^{-1})	d_{100} (Å)	q_{010} (nm^{-1})	d_{010} (Å)
PBDP-T	0	2.85	22.04	16.48	3.81
	2.5	2.83	22.20	16.38	3.84
	10	2.82	22.25	16.39	3.83
	50	2.85	22.04	16.24	3.87
PTB7-Th	0	2.61	24.08	16.09	3.91
	2.5	2.58	24.40	16.24	3.87
	10	2.55	24.64	16.34	3.85
	50	2.60	24.20	16.41	3.83
PBDB-T[a]	0	3.28	19.19	17.03	3.69
	2.5	3.15	19.95	17.13	3.67
	10	3.00	20.95	17.35	3.62
	50	3.04	20.66	17.32	3.63
PBDB-T[b]	0	2.82	22.25	17.01	3.70
	2.5	2.76	22.79	16.95	3.71
	10	2.72	23.13	17.18	3.66
	50	2.73	23.01	17.21	3.65

Note: Both lamellar and π–π stacking parameters are retrieved from the [a]out-of-plane direction and [b]in-plane direction.

Table S4. Packing parameters of (100) diffraction peaks derived from GIWAXS profiles

Polymer	$[FeCl_3]$ (mM)	q (nm^{-1})	FWHM (nm^{-1})	CCL (Å)[a]	g[b]
PBDP-T	0	2.85	0.366	154.5	0.143
	2.5	2.83	0.374	151.2	0.145
	10	2.82	0.359	157.5	0.142
	50	2.85	0.329	171.9	0.136
PTB7-Th	0	2.61	1.034	54.7	0.251
	2.5	2.58	0.844	67.0	0.228
	10	2.55	0.896	63.1	0.236
	50	2.59	0.878	64.4	0.232
PBDB-T	0	3.28	0.847	66.8	0.203
	2.5	3.15	0.800	70.7	0.201
	10	3.00	0.709	79.8	0.194
	50	3.04	0.734	77.0	0.196

Note: [a]CCL = $0.9 \times 2\pi/FWHM$; [b]$g = \sqrt{FWHM/(2\pi q)}$

Table S5. Packing parameters of (010) diffraction peaks derived from GIWAXS profiles

Polymer	[$FeCl_3$] (mM)	q (nm^{-1})	FWHM (nm^{-1})	CCL (Å)	*g*
PBDP-T	0	16.48	2.793	20.2	0.164
	2.5	16.38	2.883	19.6	0.167
	10	16.39	2.703	20.9	0.162
	50	16.24	2.883	19.6	0.168
PTB7-Th	0	16.09	3.875	14.6	0.196
	2.5	16.24	3.716	15.2	0.191
	10	16.34	3.965	14.3	0.197
	50	16.41	4.776	11.8	0.215
PBDB-T	0	17.03	3.45	16.4	0.180
	2.5	17.13	3.24	17.5	0.173
	10	17.35	3.09	18.3	0.168
	50	17.32	3.37	16.8	0.176

Note: All parameters are derived from the out-of-plane direction.

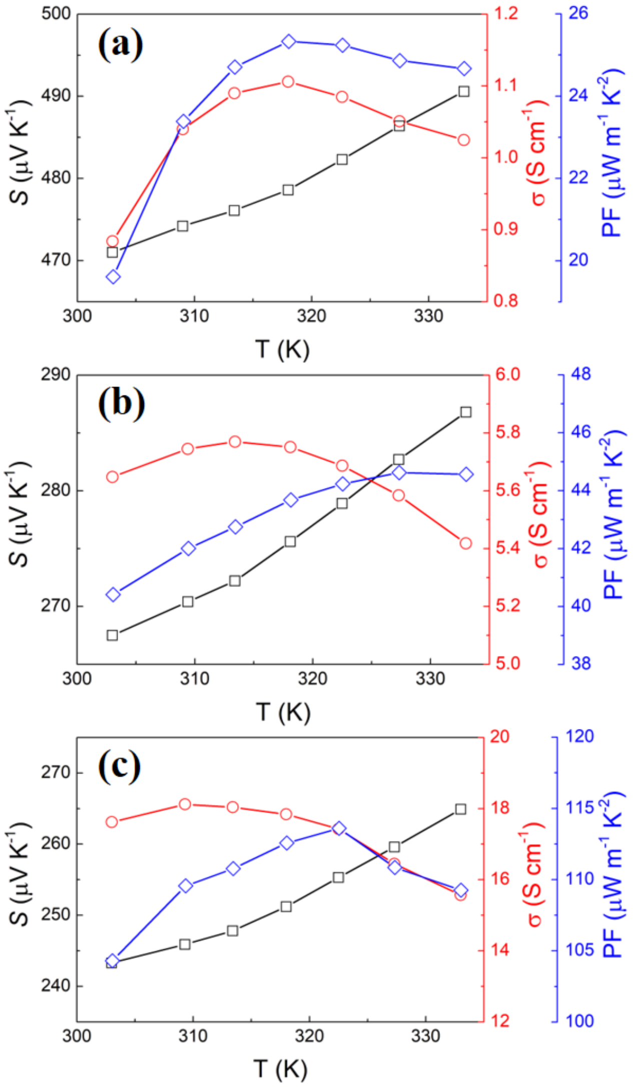

Figure S7. Temperature dependences of *S*, σ and PF for 10 mM $FeCl_3$ doped (a) PBDP-T, (b) PTB7-Th and (c) PBDB-T thin films.

Table S6. The optimal TE parameters and the corresponding carrier mobility and density of copolymers

Polymer	*S* (μV K^{-1})	σ (S cm^{-1})	PF (μW m^{-1} K^{-2})	*n* (cm^{-3})	μ_h (cm^2 V^{-1} s^{-1})
PBDP-T	488	0.85	20.1	1.88×10^{20}	0.028
PTB7-Th	487	1.94	46.0	1.90×10^{21}	0.018
PBDB-T	247	17.30	105.5	1.94×10^{21}	0.056

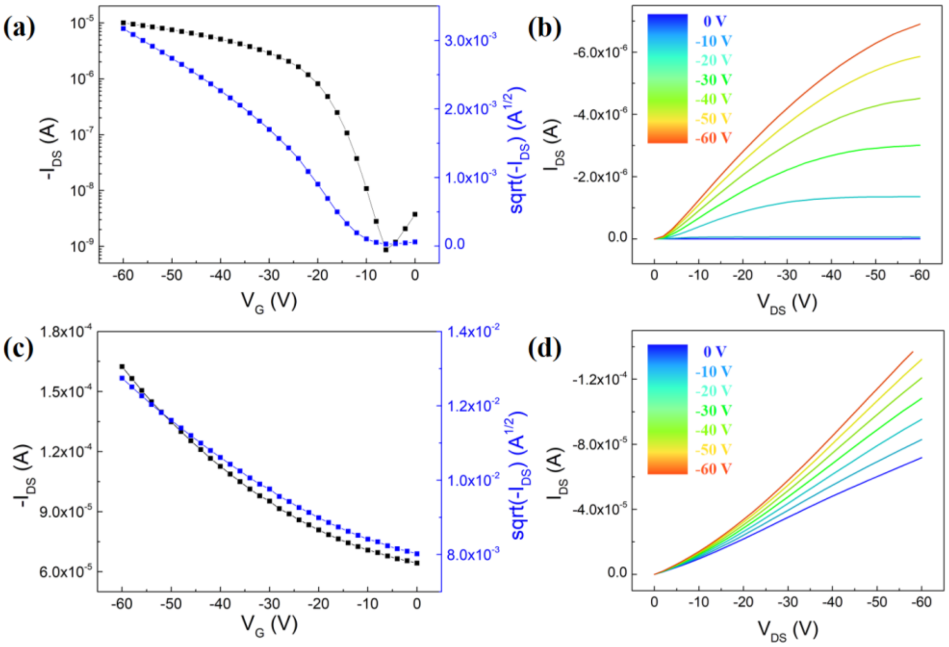

Figure S8. (a,c) Transfer and (b,d) output characteristics of (a,b) pristine PBDP-T and (c,d) $FeCl_3$ (10 mM) doped PBDP-T based bottom gate–bottom contact OFET devices.

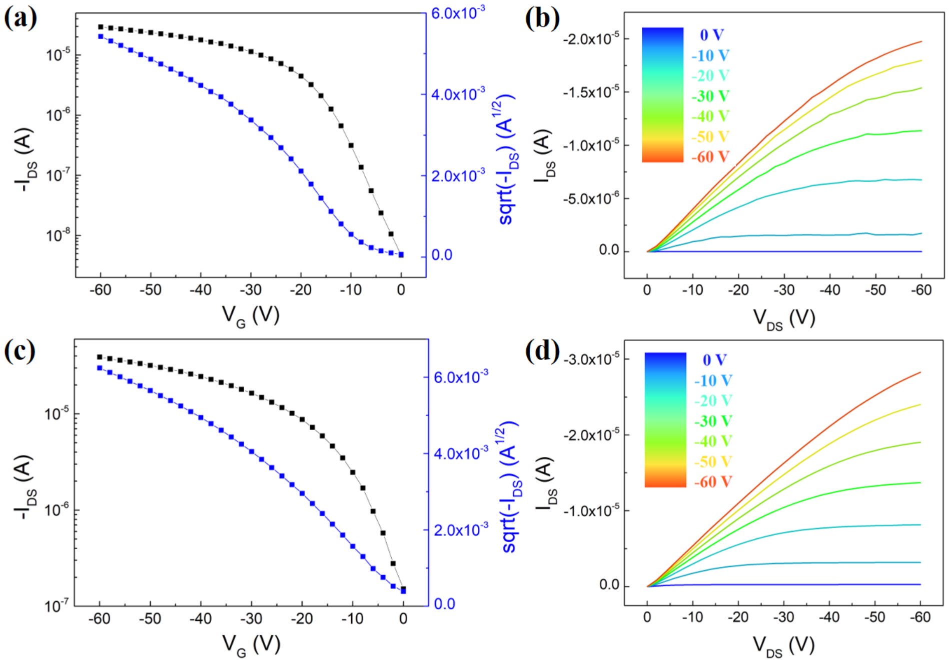

Figure S9. (a,c) Transfer and (b,d) output characteristics of (a,b) pristine PTB7-Th and (c,d) $FeCl_3$ (10 mM) doped PTB7-Th based bottom gate–bottom contact OFET devices.

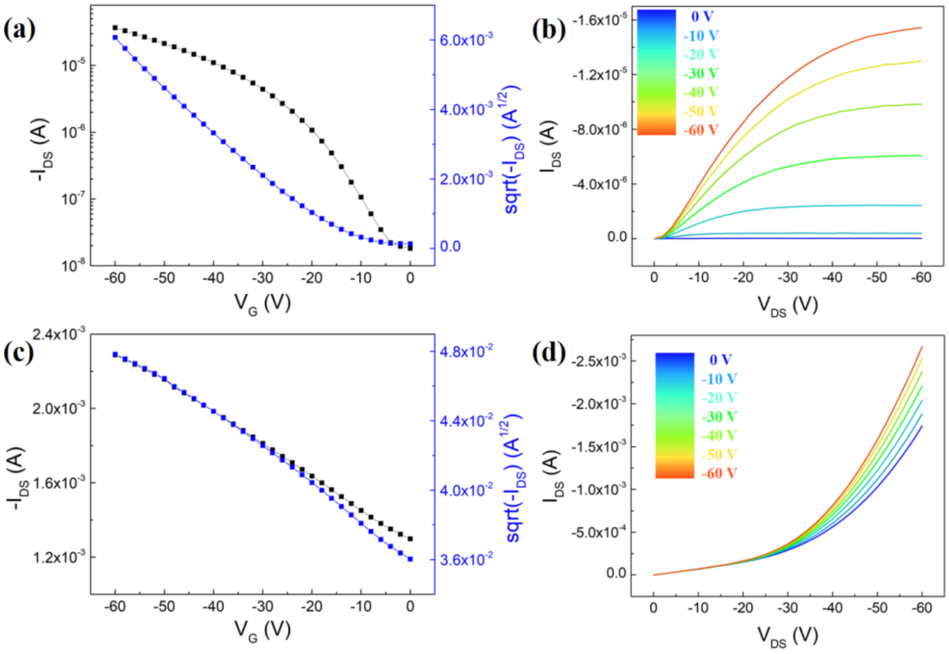

Figure S10. (a,c) Transfer and (b,d) output characteristics of (a,b) pristine PBDB-T and (c,d) $FeCl_3$ (10 mM) doped PBDB-T based bottom gate–bottom contact OFET devices.
